# Microdiamond in a low-grade metapelite from a Cretaceous subduction complex, western Kyushu, Japan

**DOI:** 10.1038/s41598-020-68599-7

**Published:** 2020-07-15

**Authors:** Tadao Nishiyama, Hiroaki Ohfuji, Kousuke Fukuba, Masami Terauchi, Ukyo Nishi, Kazuki Harada, Kouhei Unoki, Yousuke Moribe, Akira Yoshiasa, Satoko Ishimaru, Yasushi Mori, Miki Shigeno, Shoji Arai

**Affiliations:** 1 Department of Earth and Environmental Science, Graduate School of Science and Technology, Kumamoto University, 2-39-1 Kurokami, Chuo-ku, Kumamoto, 860-8555 Japan; 2 Geodynamics Research Center (GRC), Ehime University, 2-5 Bunkyo-cho, Matsuyama, 790-8577 Japan; 3 Institute of Multidisciplinary Research for Advanced Materials, Tohoku University, 2-1-1 Katahira, Aoba-ku, Sendai, 980-8577 Japan; 4 Kitakyushu Museum of Natural History and Human History, 2-4-1, Higashida, Yahatahigashi-ku, Kitakyushu, 805-0071 Japan; 5 Institute of Liberal Arts and Science, Kanazawa University, Kakuma, Kanazawa 920-1164 Japan

**Keywords:** Planetary science, Solid Earth sciences

## Abstract

Microdiamonds in metamorphic rocks are a signature of ultrahigh-pressure (UHP) metamorphism that occurs mostly at continental collision zones. Most UHP minerals, except coesite and microdiamond, have been partially or completely retrogressed during exhumation; therefore, the discovery of coesite and microdiamond is crucial to identify UHP metamorphism and to understand the tectonic history of metamorphic rocks. Microdiamonds typically occur as inclusions in minerals such as garnet. Here we report the discovery of microdiamond aggregates in the matrix of a metapelite from the Nishisonogi unit, Nagasaki Metamorphic Complex, western Kyushu, Japan. The Nishisonogi unit represents a Cretaceous subduction complex which has been considered as an epidote–blueschist subfacies metamorphic unit, and the metapelite is a member of a serpentinite mélange in the Nishisonogi unit. The temperature condition for the Nishisonogi unit is 450 °C, based on the Raman micro-spectroscopy of graphite. The coexistence of microdiamond and Mg-carbonates suggests the precipitation of microdiamond from C–O–H fluid under pressures higher than 2.8 GPa. This is the first report of metamorphic microdiamond from Japan, which reveals the hidden UHP history of the Nishisonogi unit. The tectonic evolution of Kyushu in the Japanese Archipelago should be reconsidered based on this finding.

## Introduction

The discovery of ultrahigh-pressure (UHP) minerals such as coesite and microdiamond in crustal metamorphic rocks has revolutionized our geodynamic view of the continental crust, which is considered as buoyant and therefore not able to be subducted very deeply^1–4^. The occurrence of coesite suggests a subduction depth of more than 80 km, and more than 20 coesite-bearing UHP terranes have been identified in the world. The occurrence of microdiamonds implies deeper subduction of more than 120 km depth, and at least nine well-confirmed UHP terranes containing microdiamonds are known: the Kokchetaev Massif (Kazakhstan, *ca*. 537–456 Ma)^5^, Central China (Dabie–Sulu^6^, *ca.* 240–207 Ma; Quinling^7,8^, *ca*. 490 Ma; North Qaidam^9^ *ca.*420–399 Ma), the Western Gneiss Region (Norway, *ca.* 408–375 Ma)^10^, the Ertzgebirge Massif (Germany, *ca.* 337–330 Ma)^11^, the Bohemian Massif (Czech Republic, *ca.* 340 Ma)^12,13^, the French Central Massif (*ca.* 35 – 30 Ma)^14^, the Greek Rhodope (*ca.* 65–30 Ma)^15^, Lago di Cignana in the Italian Alps (*ca.* 50–38 Ma)^16^, and the Northern Rif (Morocco, *ca.* 23–20 Ma)^17,18^. The former six terranes formed at convergent plate boundaries of Palaeozoic, the Greek Rhodope in the Cretaceous to the Palaeogene, and the latter two (the Alps and the Northern Rif) formed in the Cenozoic. Here we report the first finding of metamorphic microdiamonds from a Japanese subduction complex of the Cretaceous period, which is present at the continental margin of the China Craton.

## Geological background

**Figure 1:**
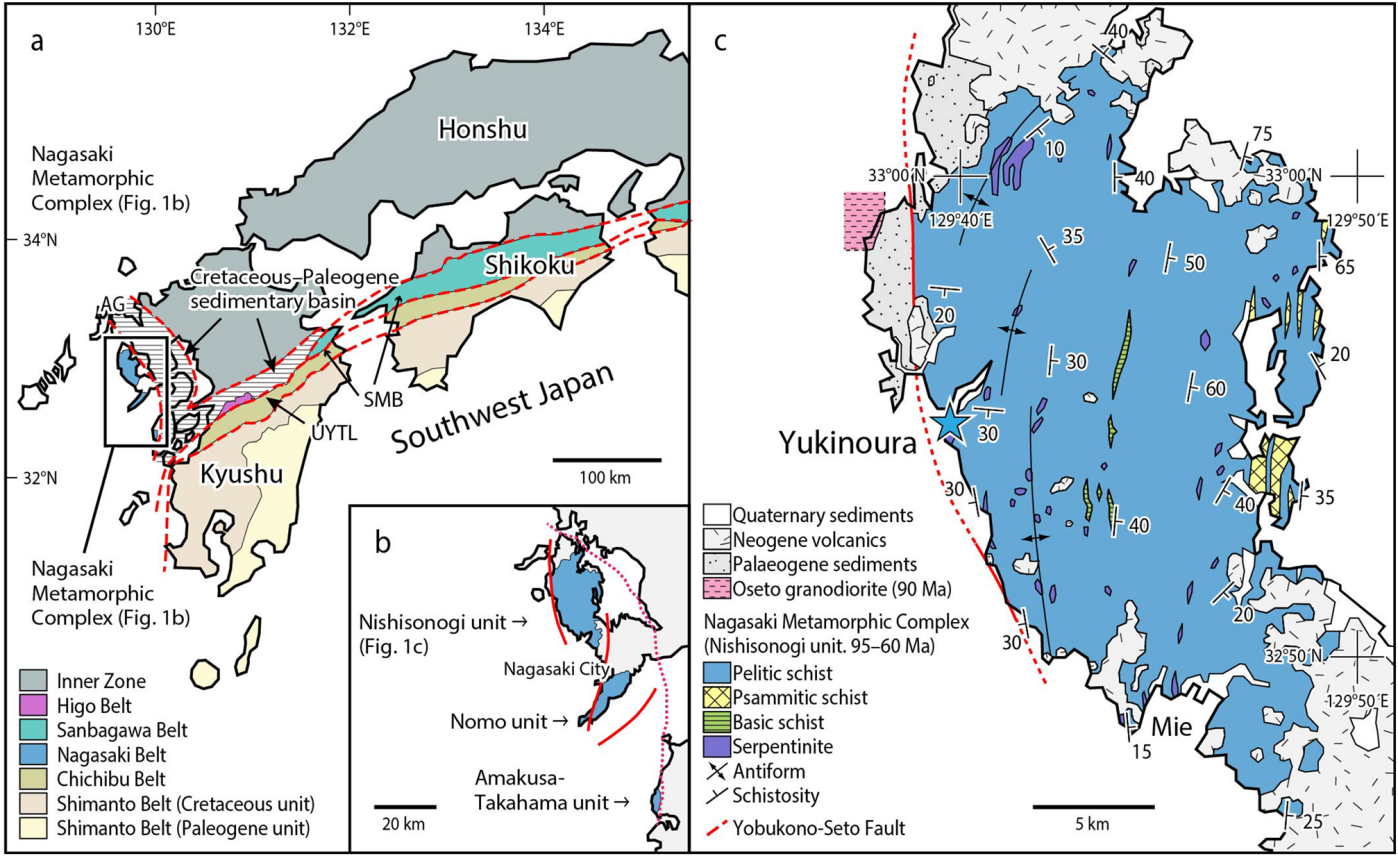
Locality of microdiamond in the Nishisonogi unit, Nagasaki Metamorphic Complex, western Kyushu, Japan. (**a**) Simplified tectonic map showing the location of the Nagasaki Metamorphic Complex together with the distribution of the Sanbagawa Metamorphic Belt (SMB). *AG* Arita graben (**b**) Map showing the distribution the Nishisonogi unit, Nomo unit, and Amakusa–Takahama unit in the Nagasaki Metamorphic Complex. (**c**) Simplified geological map of the Nishisonogi unit, showing the locality (blue star) of microdiamond (Yukinoura). Drawn with Adobe Illustaror CS6 (www.adobe.com/jp/) by Mori. Y.

The Nagasaki Metamorphic Complex (NMC) consists of high pressure (HP)–low temperature (LP) metamorphic rocks and is exposed in the Nishisonogi Peninsula (Nishisonogi unit), Nomo Peninsula (Nomo unit) and Amakusa–shimoshima Island (Amakusa–Takahama unit) in western Kyushu, Japan^19^ (Fig. 1). Microdiamonds are present in several rocks within a serpentinite mélange at Yukinoura from the Nishisonogi unit (*ca.* 95–90 Ma for glaucophane and phengite using the ^40^Ar/^39^Ar method^20^, and *ca.* 85–60 Ma by the K–Ar method^21^). The Nishisonogi unit is considered to represent an epidote–blueschist subfacies metamorphic unit that consists mainly of coherent schists (mostly pelitic and psammitic schists with minor amounts of basic schists) with minor serpentinites^19^. The serpentinite commonly occurs as a massive lenticular body, typically less than 500 m in length, which are enclosed in the coherent schists. Some serpentinite bodies are accompanied by various kinds of metabasites and minor pelitic and psammitic schists, representing the nature of serpentinite mélange^19^. The serpentinite mélange contains tectonic blocks of various lithologies and sizes embedded in a thin actinolite schist matrix or in schistose serpentinite (antigorite schist)^19^. The quartz-bearing jadeitites occur as tectonic blocks in one mélange (the Mie mélange) and exhibit a pressure and temperature of 1.5 GPa and 450 °C^19^. The Yukinoura mélange occurs at the westernmost part of the Nishisonogi unit along the Yobukono–Seto Fault (Fig. 1c) that bounds the Nishisonogi unit and the Oseto granodiorite (90 Ma)^22^. The granodiorite is overlain by the Palaeogene sediments, and there are no indications that it has thermally perturbed the Nishisonogi unit^22^.

The NMC has been correlated with the Sanbagawa Metamorphic Belt (SMB)^20,23^ in terms of similarity in the age and nature of the metamorphism^20,24^. The SMB is a high pressure (HP)–low temperature (LT) metamorphic belt of Cretaceous in age, occurring along the Median Tectonic Line in the Outer Zone of Southwest Japan^25^ (Fig. 1a). It trends east to west, and terminates at the eastern extremity of Kyushu (‘SMB’ in Fig. 1a). The NMC occurs at the western extremity of Kyushu, trending north to south (Fig. 1a). However, detailed comparison of the NMC and the SMB sheds a light on the essential differences between the two. The Nishisonogi unit is characterized by the occurrence of serpentinite mélanges involving jadeitites^19^, whereas no such serpentinite mélanges have been reported from the SMB. Eclogites are common in the SMB^26–28^, but not in the Nishisonogi unit^19^. The prograde *P–T* path of the pelitic schists in the SMB shows a linear increment in pressure and temperature from 0.6 GPa and 300 ℃ to 1 GPa and 600 ℃ based on geobarometers^29^, and the application of the Gibbs method to garnet zoning gives a steeper *P/T* path from 0.6 GPa and 470 ℃ to 0.9 GPa and 520 ℃^30^. The pelitic schists in the Nishisonogi unit show almost constant temperature of 450 ℃^31^, which is apparently lower than that in the high grade part of the SMB. The prograde *P–T* path of eclogites in the SMB goes up to 2 GPa and 600 ℃^28^ or to 3 GPa and 800 ℃^27^. No such high-grade rocks have been known from the Nishisonogi unit^19^. In the theory of a correlation between the NMC and the SMB, the main difficulty is the difference in stretching lineation trends between western Kyushu and Southwest Japan^20^. The trend of the stretching lineation is compatible to the general distribution trend of these metamorphic belts: N–S in the NMC and E–W in the SMB^25^ (Fig. 1a). Possible tectonics which caused this discrepancy (‘bending’ of the belt at western Kyushu) may be a clockwise rotation of Southwest Japan related to the opening of the Sea of Japan, during which western Kyushu remained fixed with respect to Eurasia^20^. However, all geological units in the Outer Zone of Southwest Japan such as the Chichibu Belt and the Shimanto Belt are bending southward at the southwestern part of Kyushu^32^ (Fig. 1a), extending towards the Ryukyu Arc. Therefore, it is very difficult to assume that only the SMB is bending northward. We conclude that the NMC is a geological unit independent of the SMB.

## Petrography

We have found four types of microdiamond occurrences. Type 1: inclusions in chromite from a chromitite layer in serpentinite, Type 2: inclusions in pseudotachylyte in a magnesite–quartz rock (carbonated serpentinite), Type 3: inclusions in pyrite porphyroblasts in a metapelite, and Type 4: aggregates in the matrix of a metapelite. We focus on the microdiamonds in the metapelite, Types 3 and 4, especially on the last type of microdiamond, and we will describe the former two types elsewhere. Here we describe some important lithologies related to the microdiamond.

### The microdiamond-bearing metapelite

**Figure 2:**
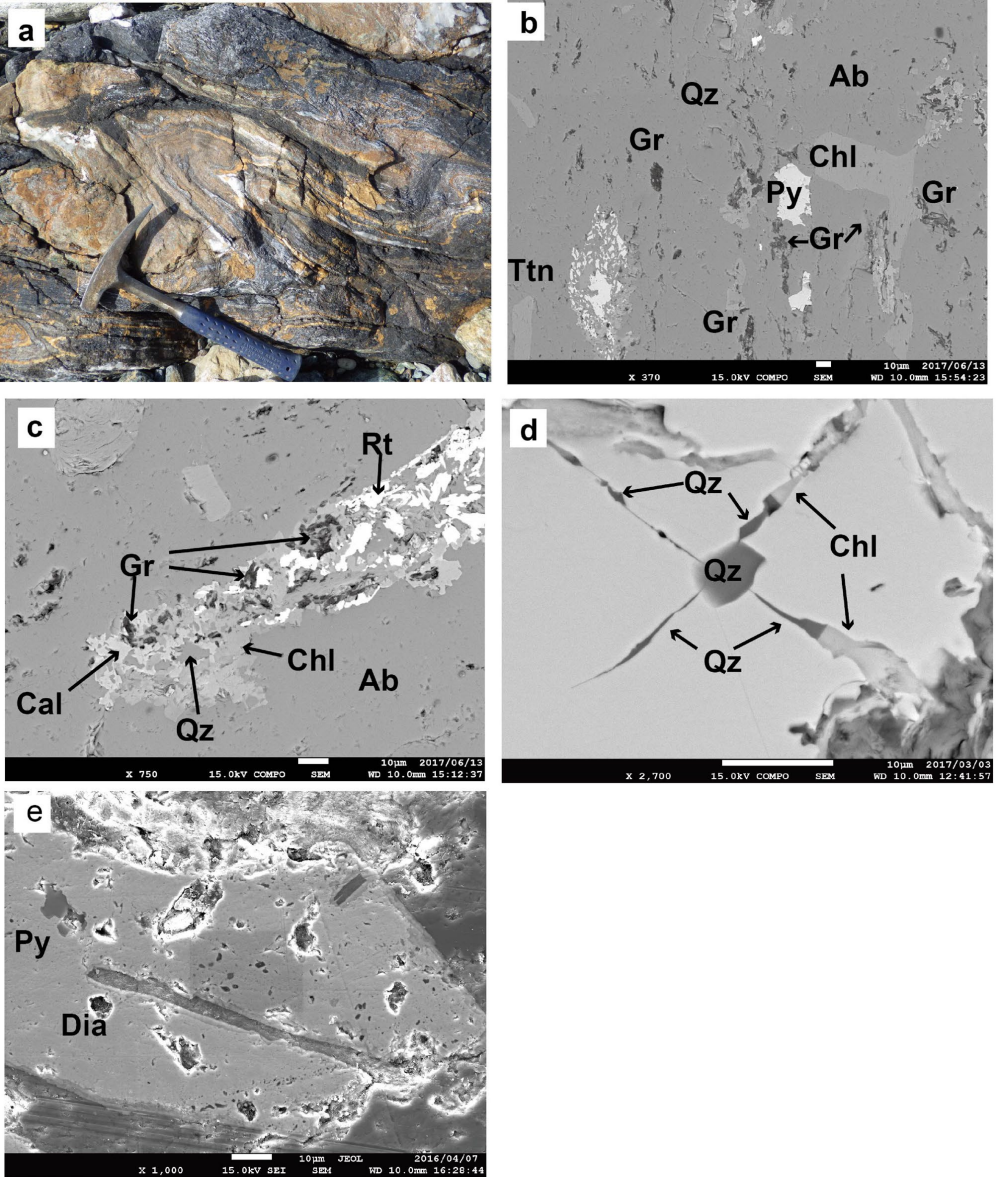
Photograph of microdiamond-bearing metapelite and back scattered electron (BSE) images of metapelite samples. (**a**) Folded metapelite which contains microdiamond. Note the development of dolomite layers (yellow) parallel to schistosity. (**b**) Texture of the metapelite showing the occurrence of graphite (Gr). Titanite (Ttn) completely decomposed into rutile, quartz (Qz) and calcite. *Ab* albite, *Chl* chlorite, *Py* pyrite. (**c**) Occurrence of graphite in the pseudomorph after titanite together with calcite (Cal), quartz, and rutile (Rt). (**d**) Quartz inclusion in garnet with radial cracks filled with quartz derived from the inclusion and with chlorite derived from the matrix. (**e**) Diamond (Dia) inclusion in pyrite.

The microdiamond-bearing metapelite is a block, several tens of meters long, in the serpentine mélange. It has a distinct feature in that dolomite layers develop parallel to the schistosity (S1) and are folded (F2) asymmetrically together with the schistosity (Fig. 2a). Later dolomite veins cut these structures obliquely. The metapelite at Yukinoura has a mineral assemblage of graphite + chlorite + phengite + albite + quartz + pyrite + dolomite + magnesite + pseudomorph after titanite (rutile and/or anatase + quartz ± calcite). Representative mineral analyses are presented in SI Table S1. Neither lawsonite nor epidote occurs in the metapelite, although epidote (or clinozoisite) is common in the coherent schists in the Nishisonogi unit. Breakdown texture of titanite (Fig. 2b,c) into rutile (and/or anatase) and quartz with or without calcite is commonly observed in all rock types involving metabasite, which will be described later, in the Yukinoura mélange. The breakdown reaction will be driven either by increase in X_CO2_ in the fluid (CO_2_-rich fluid infiltration) or by pressure increment. The pressure increment is more likely, because ubiquitous occurrence of pseudomorph after titanite in the Yukinoura mélange precludes the possibility of a local phenomenon such as fluid infiltration. Si content in phengite ranges from 3.33 to 3.43 in atoms per formula unit (*apfu*), and Mg content does from 0.48 to 0.53 *apfu* (SI Fig. S1). The empirical phengite barometer^33^ gives 1.95 to 1.99 GPa for T = 450 ^o^C^31,34^. The pressure is apparently lower than that for the graphite–diamond transition, probably because of the retrograde re-equilibration during exhumation. Garnet (Prp_3_Alm_60_Sps_20_Grs_17_ in the core and Prp_2_Alm_68_Sps_6_Grs_24_ in the rim) rarely occurs in the metapelite, and contains quartz as inclusions. Radial cracks have developed around the inclusion, and in one example cracks are filled with quartz emerging from the quartz inclusion, which are dammed by chlorite derived from the matrix (Fig. 2d). This texture strongly suggests the transformation from coesite to quartz during exhumation of the Yukinoura mélange. Pyrite occurs as strongly deformed porphyloblasts, containing inclusions of quartz, graphite and microdiamond (Fig. 2e). Microdiamond is confirmed by Raman microspectroscopy (SI Fig. S2). Electron probe microanalysis-soft X-ray emission spectroscopy (EPMA-SXES) analysis also showed a broad band derived from the sp^3^-bonded carbon of diamond (SI Fig. S3) in the spectra collected from inclusions in pyrite. These lines of evidence strongly suggest that the present mineral assemblage of the metapelite represents a retrograde one except microdiamond.

### Metabasite with a signature of retrograded lawsonite eclogite

**Figure 3:**
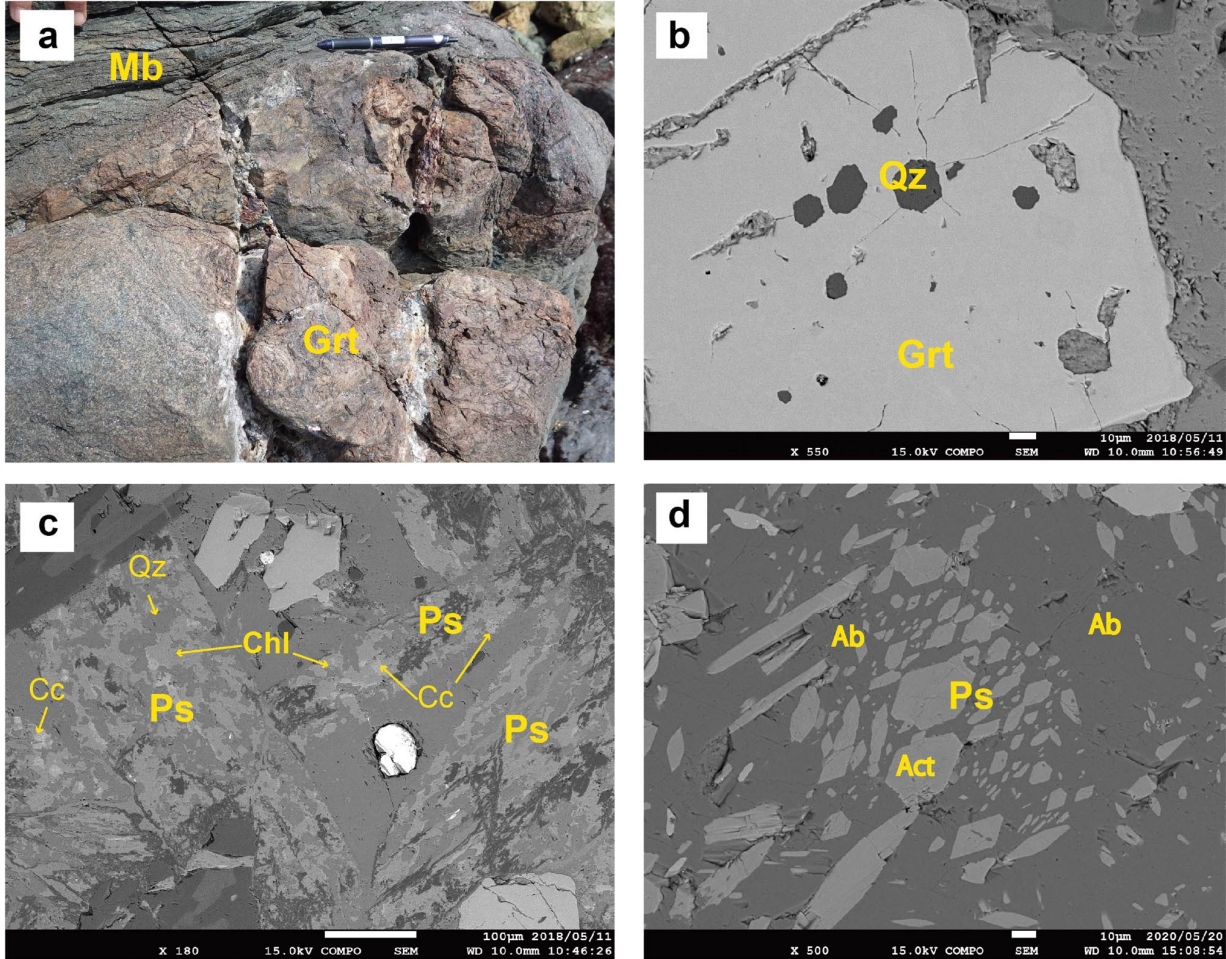
Photograph and BSE images of garnetite (Grt). (**a**) Garnetite (Grt) occurs as lenses within metabasite (Mb). (**b**) Quartz inclusion in garnet (Grt) with radial cracks. (**c**) Possible pseudomorph after lawsonite (Ps), consisting of calcite, chlorite and quartz. (**d**) Possible pseudmorph after omphacite (Ps), consisting of actinolite (Act), and albite (Ab).

Metabasite occurs as blocks several meters across in the Yukinoura mélange. It consists mainly of Ca-amphibole (actinolite or magnesio-hornblende), epidote, chlorite and albite with minor amounts of phengite, calcite, quartz, pyrite, ilmenite, and titanite. Titanite is partly or completely replaced by rutile, quartz and calcite. Garnet occasionally participates in the assemblage in various amounts. In some metabasite blocks, garnetite lenses develop in the cores of the blocks (Fig. 3a). The garnetite mainly consists of garnet, albite, chlorite (X_Mg_ = 0.25–0.45) with small amounts of Ca-amphibole (actinolite and magnesio-hornblende), epidote, quartz, green biotite (X_Mg_ = 0.42–0.45), phengite (Si = 3.41–3.72 *apfu*) and titanite (most titanites are breaking down to rutile, quartz and/or calcite). Representative mineral analyses are given in SI Table S2. Garnet contains inclusions of paragonite, glaucophane (X_Mg_ = 0.48–0.52; Fe^3+^/(Fe^3+^  + Al) = 0.17–0.38), quartz, K-feldspar, ilmenite, and apatite. Garnet shows a continuous zoning from the core (Prp_0_Alm_70–75_Sps_0–5_Grs_25–30_) to the rim (Prp_0–5_Alm_60–70_Sps_2–5_Grs_25–32_). In other samples of garnetite, garnets show different compositions richer in spessartine component such as Prp_0_Alm_30–35_Sps_30–40_Grs_25–35_ (core) to Prp_0_Alm_58–65_Sps_5–10_Grs_26–31_ (rim) and Prp_0_Alm_72_Sps_1_Grs_27_ (core) to Prp_0_Alm_21_Sps_31_Grs_48_ (rim) (SI Fig. S4). Occurrence of glaucophane, paragonite and quartz in garnet indicates a pressure condition higher than 1.5 GPa^35^. Conspicuous feature of garnet is the development of radial cracks around quartz inclusions (Fig. 3b), indicating transformation from coesite. Possible pseudomorph after lawsonite (Fig. 3c) also occurs in the garnetite. It shows a prismatic form, consisting an aggregate of chlorite, calcite, quartz with or without epidote. Possible pseudomorph after omphacite (Fig. 3d) is also found in the same garnetite, now consisting of albite, actinolite and titanite. The possible existence of lawsonite in the garnetite may suggest that lawsonite–eclogite facies is reached and that only garnet survived the retrograde metamorphism.

## Characterization of microdiamond

**Figure 4:**
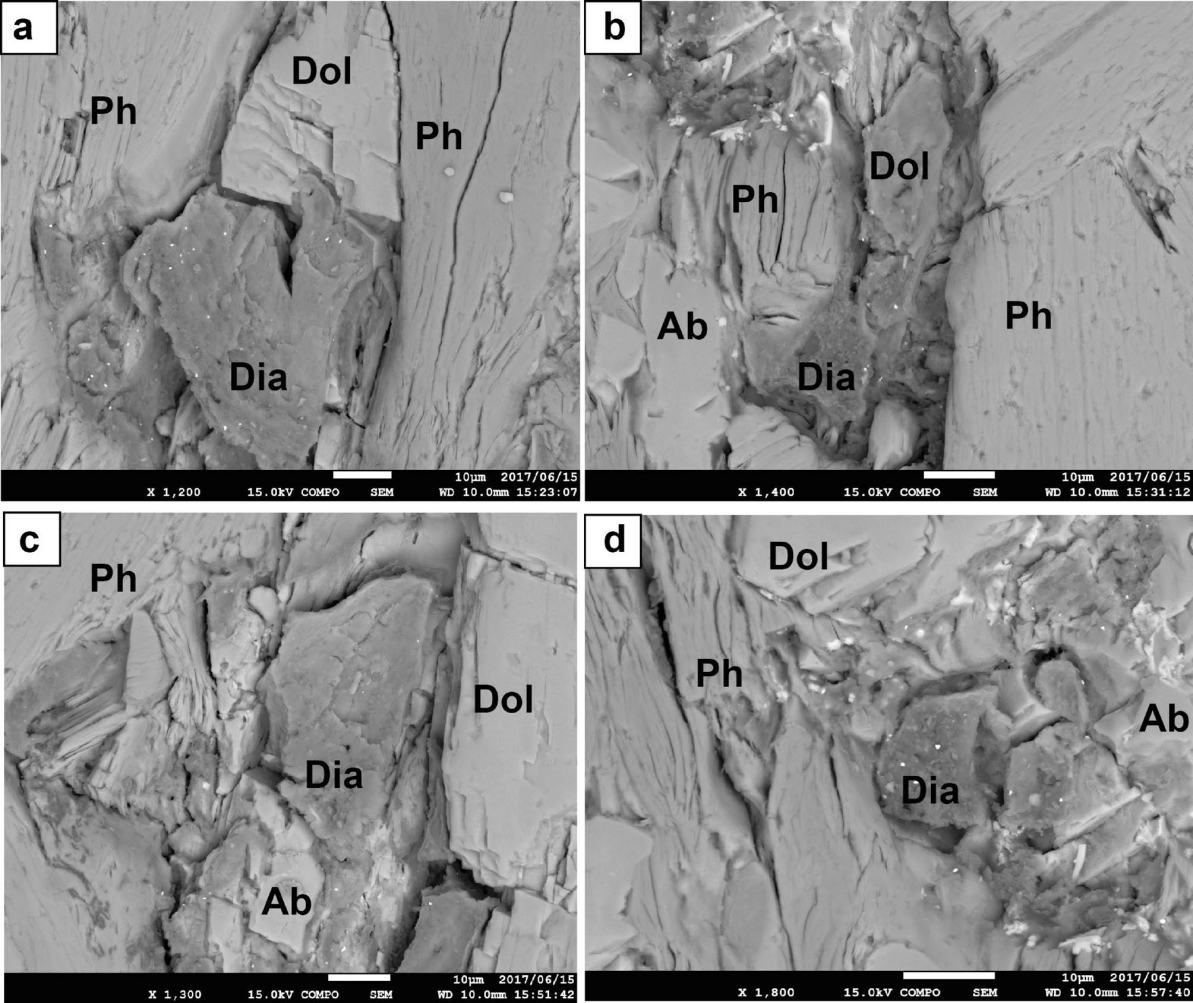
BSE images of microdiamond aggregates. (**a**–**d**) Occurrence of microdiamond aggregates of 10–50 μm in size with dolomite (Dol), phengite (Ph), and albite (Ab).

**Figure 5:**
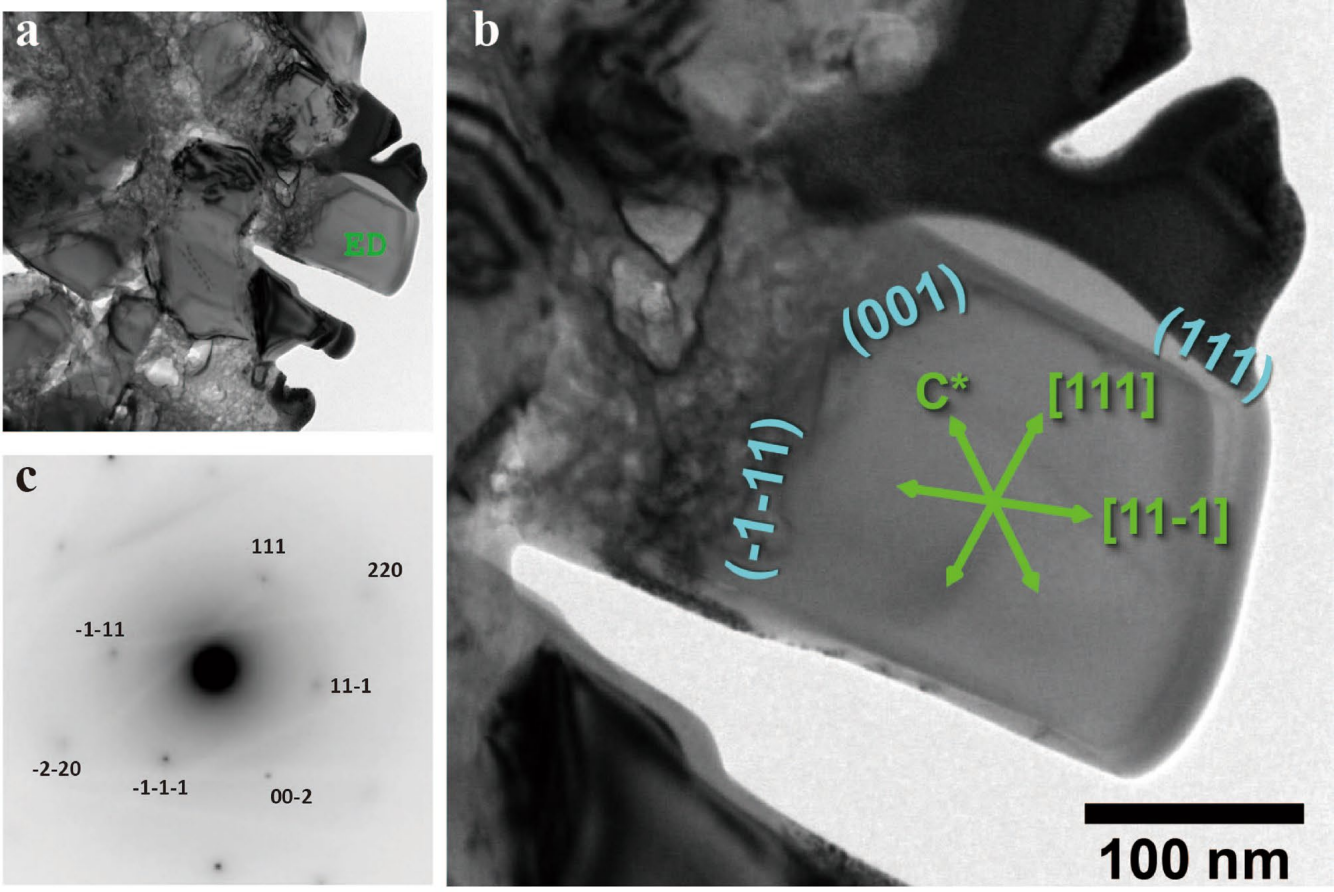
Scanning transmission electron microscope (STEM) study of microdiamond aggregate (**a**) STEM image of an aggregate of microdiamonds. (**b**) Higher magnification view. (**c**) Electron diffraction pattern obtained from a grain within the aggregate. The pattern can be explained by the reciprocal lattice along the [110] zone axis of diamond, where the *d*-spacing for the inner spot is 2.06 Å (diamond 111).

The microdiamond aggregates in Type 4 are always associated with Mg-carbonates (mostly dolomite and occasionally magnesite) and show irregular shapes of 10–50 μm in size that consist of numerous diamond grains embedded in phengite (Fig. 4a–d). The phengite is very fine-grained (~ 500 nm in size), and is confirmed with scanning transmission electron microscopy–energy dispersive X-ray spectroscopy (STEM–EDS) and by the electron diffraction method. Each diamond crystal is subhedral to euhedral and 0.3–0.6 μm in diameter (Fig. 5a,b). The selected area electron diffraction (SAED) pattern is obtained from a grain (Fig. 5c) within the aggregate. It can be reasonably explained by the reciprocal lattice along the [110] zone axis of diamond, where the *d*-spacing of the inner spot is 2.06 Å (diamond 111). In addition, this diamond crystal seems to show a euhedral (cubo-octahedral) morphology terminated by {100} and {111} facets. STEM–EDS analysis of the aggregates shows a strong peak due to C together with peaks for Si, Mg, and Al, which reflect the matrix (phengite) composition. Raman microspectroscopy measurements of the aggregates shows a strong band at 1,332 cm^−1^, with a full width at half maximum (FWHM) of ~ 5 cm^−1^, which corresponds to the T_2g_ mode for diamond (SI Fig. S5a). Most aggregates do not show any graphite bands, with two exceptions; one shows a weak and broad D1 band (1,350 cm^−1^) due to disordered graphite associated with a strong band of diamond at 1,332.5 cm^−1^ (SI Fig. S5b), and the other shows a diamond band at 1,316 cm^−1^ with a broad G band of graphite at around 1,570 cm^−1^ (SI Fig. S5c). These lines of evidence clearly indicate the presence of natural microdiamonds.

## Discussion

### Temperature condition estimated by Raman microspectroscopy of graphite

The peak metamorphic temperature of the metapelite was estimated to be 450 °C according to Raman microspectroscopy of graphite^31,34^. Graphite shows two kinds of occurrence. The first type of graphite occurs along the cleavages of chlorite and phengite and also occurs as inclusions in albite and in quartz (Fig. 2b). The second type of graphite occurs as tiny grains in pseudomorphs after titanite (Fig. 2c). Only graphite inclusions in albite and in quartz (graphite grains beneath the surface of a transparent mineral) are measured by Raman microspectroscopy to estimate the formation temperature of graphite^31,34^, because graphite exposed on the surface of thin section (e.g., the case of the second type) is damaged by polishing to give lower temperatures^36^. The first type of graphite may have originated from carbonaceous matter in the original sediments, because it occurs along the cleavages of chlorite and phengite and also as inclusions in albite and in quartz. The second type of graphite may have precipitated from a C–O–H fluid during the breakdown of titanite into rutile + quartz + calcite. The first type of graphite may be a product of prograde graphitization, which is an irreversible reaction^37^. Therefore, the temperature estimated for the first type of graphite by Raman microspectroscopy can be regarded as a peak metamorphic temperature. Laboratory deformation experiments showed that the mechanical modification of graphite structure owing to deformation makes the estimation of metamorphic temperature by Raman microspectroscopy ambiguous^38^. However, graphite inclusions in albite and quartz can escape such effects of strong deformation; they will give a meaningful metamorphic temperature. The graphite could easily persist to diamond stability field without overcoming the barrier of activation energy to convert to diamond. If the formation temperature for the present microdiamond is the same as that of graphite (450 °C), the pressure condition will be higher than 2.8 GPa based on the graphite–diamond equilibrium (SI Fig. S6).

### Genesis of microdiamond

Such aggregates of microdiamonds described above are rare in ultrahigh-pressure metamorphic (UHPM) terranes. Compared to polycrystalline microdiamonds reported from Kokchetav^5^ and also from Erzgebirge^11^, the Yukinoura microdiamonds occur as aggregates of many single crystalline microdiamonds. The microdiamonds have the following distinct features. Firstly, the size of each microdiamond grain (0.2–0.6 μm) is much smaller than that (10–80 μm) of microdiamonds from other UHPM terranes^3^. Secondly, Yukinoura microdiamond has almost euhedral cuboid or octahedral forms, whereas those reported to date from UHPM terranes show a wide range of morphologies from skeletal, rose-like, and hopper to perfect euhedral shapes^3^. The morphology of diamond is dependent on the ratio of the driving force for nucleation and growth to the reaction kinetics^41^, and the latter is dependent on several factors, such as the temperature, the fluid composition, and the presence of melt. High temperature and the presence of melt facilitate the formation of euhedral diamond^41^; however, that is not the case in Yukinoura. The coexistence of Mg-carbonates strongly suggests the presence of a C–O–H fluid. The C–O–H fluid may play a crucial role for the formation of highly crystalline microdiamond, even at low temperature, as shown in the case of Lago di Cignana (Western Alps, Italy), in which microdiamonds occur as inclusions in garnet formed at approximately 600 °C^16^. In the case of the Maksyutov Metamorphic Complex, South Ural Mountains^42^, the poor crystallinity is compatible with relatively low temperature, solid state growth in the absence of both melt and a C–O–H–N fluid^42^. The contrast between the two cases of low-temperature microdiamond clearly shows the potential importance of fluid in determining the crystallinity and morphology of microdiamond.

The mechanism for the formation of microdiamond in metapelites is an interesting issue to be discussed. The possibility of detrital origin of diamond can be precluded, because microdiamonds will be decomposed into graphite during the prograde metamorphism. Conversely, microdiamonds can be preserved during the retrograde metamorphism because the activation energy may not be available for the transformation to graphite. Therefore, our discussion will be based on an assumption of the metamorphic origin of the microdiamonds. First, we discuss the meaning of the formation temperature of graphite estimated by Raman microspectroscopy and the relation between microdiamond and graphite. The first type of graphite may have originated from carbonaceous matter in the original sediments, because it occurs along the cleavages of chlorite and phengite and also as inclusions in albite and in quartz. The second type of graphite may have precipitated from a C–O–H fluid during the breakdown of titanite into rutile + quartz + calcite. The first type of graphite will be a product of prograde graphitization, which is an irreversible reaction^37^. Therefore, the temperature estimated for the first type of graphite by Raman microspectroscopy can be regarded as a peak metamorphic temperature. It is experimentally confirmed that hexagonal diamond (lonsdaleite) stably formed from well crystallized graphite by solid state transformation only at temperatures above 1,000 ^o^C^43^. Therefore, graphite could easily persist to diamond stability field without converting to diamond in low-grade metamorphic rocks. If the formation temperature for the present microdiamond is the same as that of graphite (450 °C), the pressure condition will be higher than 2.8 GPa based on the graphite–diamond equilibrium. Such conditions correspond to the lawsonite eclogite facies^4^ (see SI Fig. S3). The first question is the possibility of the metastable growth of microdiamond in the stability field of graphite. Simakov^44,45^ showed that nanosized diamond can form from a C–O–H fluid under low pressures in the graphite stability field. Manuella^46^ theoretically examined the metastable growth of diamond in serpentinite-hosted hydrothermal systems based on the Laplace–Young Equation^47^, which gives the additional curvature-induced pressure associated with the formation of spherical and quasi-isotropic nanocrystals. The size-dependent internal pressure *Pa* is defined as *Pa* = 4γ/*d*, where γ stands for surface free energy and *d* does size (nm). The transition pressure *P* decreases by *Pa*: *P* (GPa) = 0.003* T* (K) + 0.4171 – *Pa*. The size of metastable diamond is determined by the following equation: *d* = –14.8/(*P* − 0.003* T* − 0.4171)^47^. Based on this theory, Manuella^46^ showed that the size of diamond will be smaller than 6 nm at temperatures between 700 and 1200 K. The Yukinoura microdiamonds are much larger (0.2–0.6 μm) than this; therefore, they are unlikely to have formed metastably. If Yukinoura microdiamonds formed in the diamond stability field, then the pressure would be as high as at least 2.8 GPa, which corresponds to the lawsonite–eclogite facies as mentioned above. We have several lines of evidence supporting high pressure (HP) to UHP condition: (1) pseudomorph after coesite (Figs. 2d, 3b), (2) high Si content in phengite as described in *Petrography*, and (3) pseudomorph (rutile + quartz ± calcite) after titanite (Fig. 2b,c), and (4) the possibility of nearby lawsonite–garnet assemblages (Fig. 3).

A possible mechanism for the stable formation of microdiamond could be direct precipitation from a C–O–H fluid that consists of neutral species such as CO_2_, CH_4_, H_2_ and H_2_O, in response to a change of oxygen fugacity^16^. In the case of Lago di Chignana Unit (LCU) in the Alps^16^, microdiamonds occur as inclusions in spessartinous garnet together with inclusions of dolomite and magnesite and with H_2_O-rich fluid inclusions. Based on the finding of Fe^3+^ enrichment in garnet around diamondiferous inclusions, a redox reaction between the trapped fluid and garnet is proposed^16^ to explain simultaneous formation of microdiamond and Fe^3+^-rich garnet such as: 2CaFe(CO_3_)_2_ in dolomite + CaCO_3_ + 3SiO_2_ = Ca_3_Fe_2_^3+^Si_3_O_12_ + 3C + 3/2O_2_. A thermodynamic modelling of C–O–H fluid equilibria by the same authors indicates possible precipitation of graphite or diamond from the fluid within each stability field along a prograde path, under an assumption of redox equilibrium between the host rock and the fluid^16^. Graphite occurrence in pseudomorphs after titanite (Fig. 2c) in the Yukinoura metapelites is very consistent with this model, and diamond precipitation from fluid may also be explained by the same mechanism.

### Implications on the tectonics of the Southwest Japan

**Figure 6:**
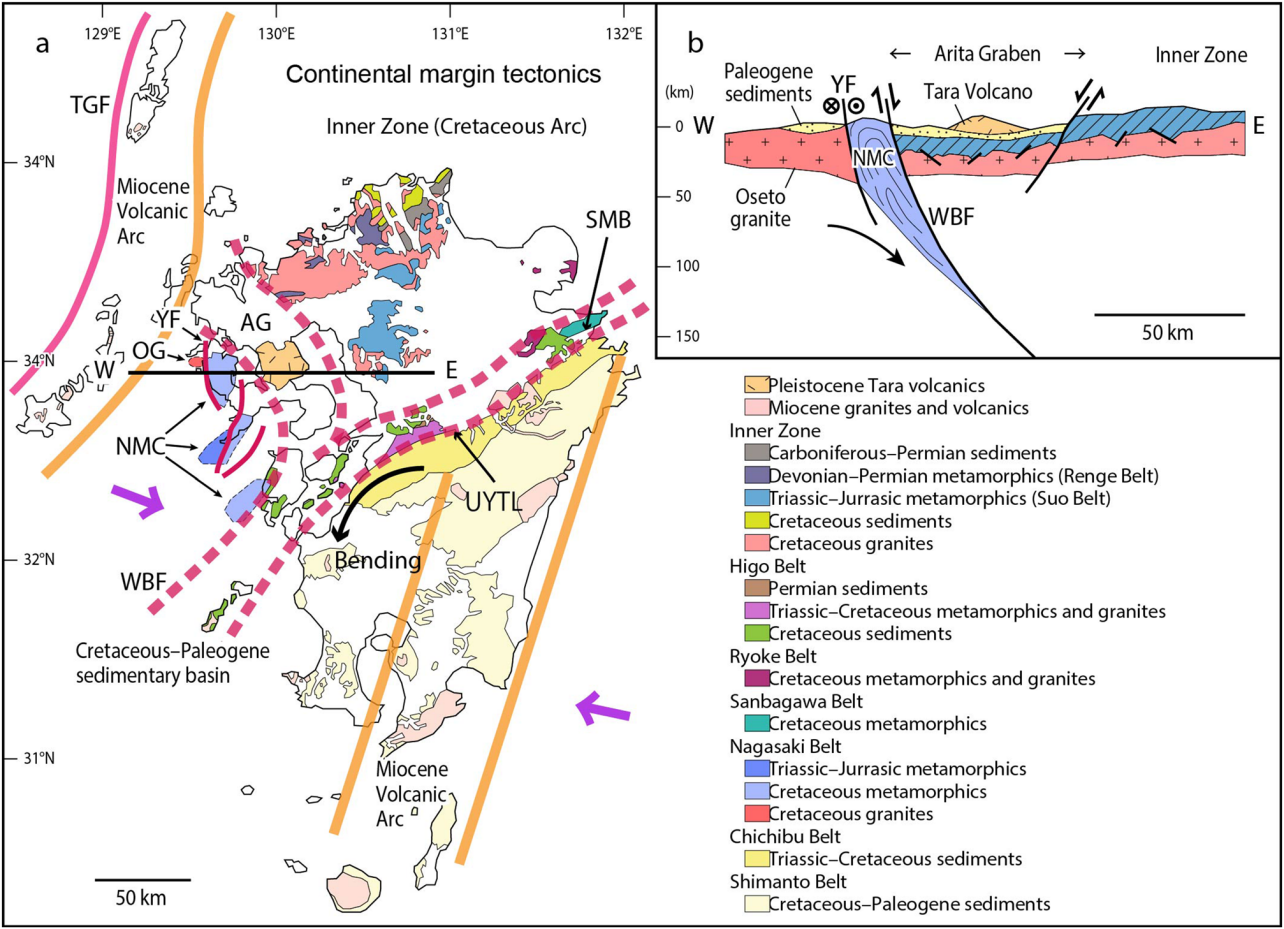
Schematic geotectonic map of Kyushu showing the distribution of basement rocks. Cenozoic volcanic rocks and sedimentary formations are not shown. (**a**) Kyushu is divided into the Inner Zone, Outer Zone and the westernmost part (the Nagasaki Metamophic Complex: NMC) by a graben along the Usuki–Yatsushiro Tectonic Line (UYTL) and also by the Arita graben (AG). Two Miocene volcanic arcs are shown by thick orange lines. Purple arrows show possible subduction directions. (**b**) Cross section along the line W–E in (**a**). *YF* Yobukono–Seto fault, *OG* Oseto granodiorite, *TGF* Tsushima–Goto Fault, *WBF* western border fault of the Arita graben, *SMB* Sanbagawa Metamorphic Belt. Drawn with Adobe Illustaror CS6 (www.adobe.com/jp/) by Mori. Y.

This is the first report of the occurrence of microdiamonds from a palaeo-subduction complex in Japan, which urges reconsideration of geotectonic evolution of the Japanese Archipelago. Kyushu is divided into the Inner Zone (northern part) and Outer Zone (southern part) by the Usuki–Yatsushiro tectonic line (‘UYTL’ in Fig. 6), along which a narrow sedimentary basin of the Cretaceous to the Palaeogene develops^23^. It merges with the Arita graben, trending north to south, at the east of the NMC. The Arita graben is mostly covered by Quaternary volcanic rocks (basalt and dacite with minor rhyolite) with a Palaeogene basement^48^. The NMC is separated by the graben, both from the Inner Zone and from the Outer Zone^23^. To the west of the NMC, the Oseto granodiorite (‘OG’ in Fig. 6) of *ca*. 90 Ma occurs in fault contact with the NMC, and has not had a thermal effect on the NMC^22^. The fault is named the Yobukono–Seto fault^22^ (‘YF’ in Fig. 6), which is a dextral strike slip fault. To the west of the Oseto granodiorite, a Miocene volcanic arc developed in a north to south orientation^23^. A volcanic arc of the same age also occurs along the southeast part of Kyushu^23^. The parallel alignment of the two Miocene volcanic arcs suggests that the tectonics of Kyushu is not solely governed by the subduction of the Philippine Sea Plate. The NMC may have formed along a narrow zone between the Chinese craton and Kyushu by subduction of the marginal part of the Chinese craton, and displaced along the strike-slip fault system involving the Tsushima–Goto fault (‘TGF’ in Fig. 6) and the Yobukono–Seto fault during the opening of the Sea of Japan^48^. Although the lack of geophysical data such as seismic tomography in this region prevents us to construct a detailed tectonic model involving the underground structure, we will briefly discuss a possible tectonic model which may explain the development of the UHP unit in the western part of Kyushu. Guillot et al.^49^ proposed three types of subduction regime to explain different styles of exhumation: the accretionary-type, the serpentinite-type and the continental-type. The Nishisonogi unit may represent the coexistence of an accretionary wedge and a serpentinite subduction channel in a single subduction zone^49^, because the unit consists mainly of metasedimentary rocks with several thin (less than 350 m) and elongated bodies of serpentinite melanges^19^. The existence of a decoupling zone within the upper part of the subducting slab is essential for the exhumation of HP to UHP rocks, because only slices of the upper decoupled part will be exhumed in contrast to the downgoing major part of the slab^50^. The serpentinite mélange may have acted as a decoupling zone in the case of the Nishisonogi unit. According to the numerical simulation^51^, the evolution of the subduction zone consists of two distinct stages: (1) the early stage without return flow from depths exceeding about 20 km and (2) the mature stage with intense return flow from greater depths. The hydration of the mantle wedge will control the transition between these two stages. The hydration of the hanging wall mantle may cause the widening of the subduction channel, resulting in the onset of forced return flow^51^. The serpentinite mélange will form by this forced return flow and is subsequently exhumed to the surface. Among several factors necessary for exhumation of HP to UHP metamorphic rocks^49^, thrusting associated with normal faulting and return flow in the subduction channel may be the important factors in the case of the Nishisonogi unit. The western border fault (‘WBF’ in Fig. 6) of the Arita graben will be a normal fault, and the Yobukono–Seto fault will be a thrust with a dextral slip component. At the mature stage of the subduction zone, the exhumation of the Nishisonogi unit may have been triggered by forced return flow owing to the hydration of hanging wall, and serpentinite mélanges may have acted as a detachment zone which enhanced the exhumation. Simultaneous activities of the western border fault of the Arita graben and the Yobukono–Seto fault were possibly associated with the return flow. As a result, the serpentinite mélange could have exhumed from the depth of UHP condition. Thus, finding of microdiamonds from the Yukinoura mélange unravels the hidden UHP history of the Nishisonogi unit, the Nagasaki Metamorphic Complex, which has been considered as an epidote–blueschist subfacies metamorphic unit until this finding.

## Methods

Polished petrological thin sections were prepared with Al_2_O_3_ lapping sheets (Sankyo Rikagaku Co. Ltd.) on a grooved glass plate to avoid contamination of diamond from diamond paste, after grinding with SiC. The sections were coated with Pt for SEM–EDS analysis. The same sections were used for micro-Raman spectroscopy.

Raman spectra were measured with a Raman spectrometer (Horiba Jovin Yvon LabRAM HR800) equipped with a diode-pumped solid state laser (Fandango 50, 514.5 nm) at Kumamoto University. A silicon standard was used for calibration before each measurement. The laser power was set at 50 mW on the sample surface, and the laser was focused on the sample using a microscope (Olympus BX41) with a 100 × objective lens (LMPlan FL: NA = 0.80). The operating conditions were a 1,000 μm pinhole, a 25 μm slit, an 1,800 line/mm grating, and an acquisition time of 30 s.

Mineral chemistry was determined with a SEM–EDS system (JEOL JSM-7001F field emission scanning microscope equipped with an Oxford Aztec energy-dispersive X-ray spectrometer) operating at an accelerating voltage of 15 kV with a beam current of 1.02 nA.

A focused ion beam (FIB) system (FEI, Scios) was used to prepare transmission electron microscopy (TEM) foils (typically, 15 × 10 × 0.1 μm) from the target areas marked by the micro-Raman and FE-SEM examinations. Prior to milling, the target area was coated with a Pt layer (~ 1 μm thick) using a gas injection system equipped on the FIB to protect the outermost surface from the ion beam. TEM observations were performed using a microscope (JEOL JEM-2100F) operated at 200 kV and equipped with CCD cameras (Gatan Orius 200D and UltraScan 1,000) and an EDS detector (JEOL JED-2300T). Bright-field imaging and selected area electron diffraction (SAED) were used for microtexture observation and phase identification. Scanning TEM (STEM) imaging and chemical analysis with EDS were conducted using a focused electron beam (with a spot size of 0.5 nm) and a camera length of 40 cm.

Soft X-ray emission spectroscopy (SXES) based on electron microscopy can be used to identify the chemical bonding state of small specimen areas. 2nd order C K-emission spectra were obtained using a commercial SXES instrument (SS-94000SXES) attached to an electron probe microanalyzer (EPMA; JEOL JXA-8230). Experimental conditions such as the accelerating voltage, probe current and acquisition time were 5 kV, 20 nA and 20 s, respectively. The specimen volume examined was approximately 1 μm in diameter.

## Supplementary information

Supplementary file1 (PDF 550 kb)
