# Supplementary material for: Microdiamond in a low-grade metapelite from a Cretaceous subduction complex, western Kyushu, Japan
Source: Sci Rep. 2020 Jul 15;10:11645. doi: 10.1038/s41598-020-68599-7 (PMC7363827; doi:10.1038/s41598-020-68599-7)
Supplement: Supplementary file 1 — Supplementary file1 (PDF 550 kb) [file 41598_2020_68599_MOESM1_ESM.pdf]

Supplementary information for:

Microdiamond in a low-grade metapelite from a Cretaceous subduction complex, western Kyushu, Japan

Tadao Nishiyama[1], Hiroaki Ohfuji[2], Kousuke Fukuba[2], Masami Terauchi[3], Ukyo Nishi[1], Kazuki Harada[1], Kouhei Unoki[1], Yousuke Moribe[1], Akira Yoshiasa[1], Satoko Ishimaru[1], Yasushi Mori[4], Miki Shigeno[4], and Shoji Arai[5]

[1] Department of Earth and Environmental Science, Graduate School of Science and Technology, Kumamoto University, 2-39-1 Kurokami, Chuo-ku, Kumamoto 860-8555, Japan

[2] Geodynamics Research Center (GRC), Ehime University, 2-5 Bunkyo-cho, Matsuyama 790-8577, Japan

[3] Institute of Multidisciplinary Research for Advanced Materials, Tohoku University, 2-1-1 Katahira, Aoba-ku, Sendai 980-8577, Japan

[4] Kitakyushu Museum of Natural History and Human History, 2-4-1, Higashida, Yahatahigashi-ku, Kitakyushu 805-0071, Japan

[5] Institute of Liberal Arts and Science, Kanazawa University, Kakuma, Kanazawa 920-1164, Japan

Correspondence and requests for materials should be addressed to T.N.
(e-mail; tadaonishiyama@gpo.kumamoto-u.ac.jp)

This file contains:
SupplementaryTables 1-2
Supplementary Figures S1-S4

Supplementary Table1. Representative analyses of minerals in the metapelite

Mineral	Grt core	Grt rim	Ph	Ph	Ph	Chl	Ab
SiO_2 (wt%)	36.00	36.49	49.75	49.35	49.87	27.32	67.85
Al_2O_3	20.52	20.69	26.85	26.22	26.46	22.35	19.18
FeO*	28.08	31.01	3.45	4.08	4.03	24.78	-
MnO	7.71	2.47	-	-	-	0.53	-
MgO	0.54	0.71	2.37	2.61	2.59	16.52	-
CaO	6.97	8.45	-	-	-	-	-
Na_2O	-	-	0.3	-	-	-	11.91
K_2O	-	-	9.83	9.7	9.67	-	-
total	99.82	99.82	92.55	91.96	92.62	91.50	98.94
O	12	12	11	11	11	28	8
Si (apfu)	2.95	2.96	3.42	3.43	3.43	5.46	3.00
Al	1.98	1.98	2.18	2.15	2.15	5.26	1.00
Fe^{2+}	1.92	2.11	0.2	0.24	0.23	4.14	-
Mn	0.53	0.17	-	-	-	0.09	-
Mg	0.07	0.09	0.25	0.27	0.27	4.92	-
Ca	0.61	0.74	-	-	-	-	-
Na	-	-	0.04	-	-	-	1.02
K	-	-	0.86	0.86	0.85	-	-
Σcation	8.06	8.05	6.95	6.95	6.93	19.87	5.02

Abbreviations: Grt, garnet; Ph, phengite; Chl, chlorite; Ab, albite; apfu, atoms per formula unit

FeO*: total iron as FeO

Supplementary Table2. Representative analyses of minerals in the garnetite

Mineral	Grt core	Grt rim	Gln	Ca-amph	Bt	Ph	Pg
SiO_2 (wt%)	36.17	37.00	55.78	51.45	35.02	50.71	41.80
TiO_2	-	-	-	-	0.94	-	-
Al_2O_3	20.08	20.56	11.05	4.18	16.27	23.97	40.10
FeO*	32.77	28.99	18.76	15.42	22.97	3.72	1.10
MnO	1.00	-	-	-	0.46	-	-
MgO	0.72	1.10	5.76	13.19	9.91	3.00	-
CaO	8.68	11.63	0.45	10.81	-	-	1.40
Na_2O	-	-	6.62	1.42	-	-	6.50
K_2O	-	-	-	-	6.96	9.63	0.30
total	99.42	99.28	98.42	96.47	92.53	91.03	91.20
O	12	12	23	23	11	11	11
Si (apfu)	2.93	2.97	7.86	7.52	2.77	3.54	2.81
Ti	-	-	-	-	0.06	-	-
Al	1.92	1.95	(Al^{IV}) 0.14	(Al^{IV}) 0.48	1.52	1.98	3.18
			(Al^{VI}) 1.70	(Al^{VI}) 0.24			
Fe^{3+}	0.15	0.06	0.24	0.45	-	-	-
Fe^{2+}	2.08	1.89	1.97	1.44	1.52	0.22	0.06
Mn	0.07	-	-	-	0.03	-	-
Mg	0.09	0.13	1.21	2.87	1.17	0.32	-
Ca	0.75	1.00	0.07	1.69	-	-	0.1
Na	-	-	1.81	0.40	-	-	0.85
K	-	-	-	-	0.70	0.86	0.03
Σcation	7.99	8.00	15.00	15.10	7.77	6.92	7.03

Abbreviations: Grt, garnet; Gln, glaucophane; Ca-amph, Ca-amphibole; Bt, biotite; Ph, phengite; Pg, paragonite; apfu, atoms per formula unit

FeO*: total iron as FeO

Ferric estimations: total cation = 15.0 for Gln and total cation - Ca = 13 for Ca-amph.

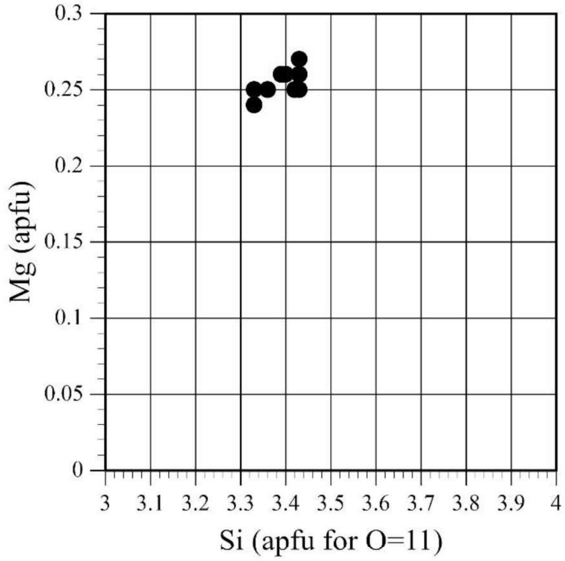

Supplementary Figure S1. Compositions of phengite from the metapelite in the plot of Mg (atoms per formula unit: apfu) vs. Si.

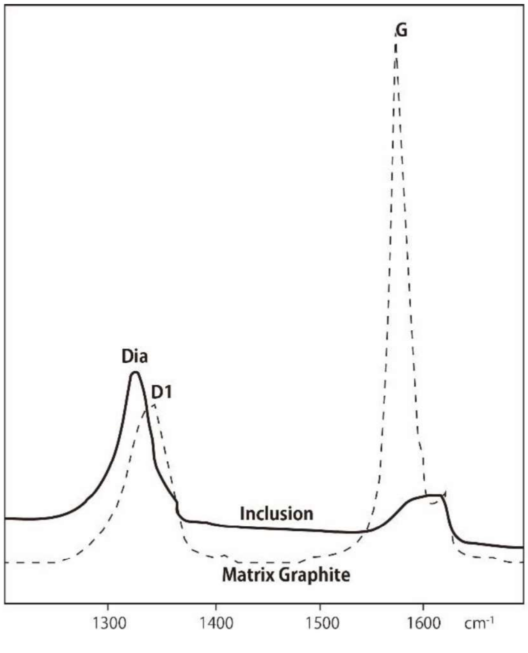

Supplementary Figure S2. Raman spectrum of microdiamond inclusion in pyrite and that of graphite in the matrix of the same metapelite. Dia: diamond band at 1330 cm^{-1}, D1: D1 band of graphite at 1350 cm^{-1} and G: G band of graphite at 1580 cm^{-1}.

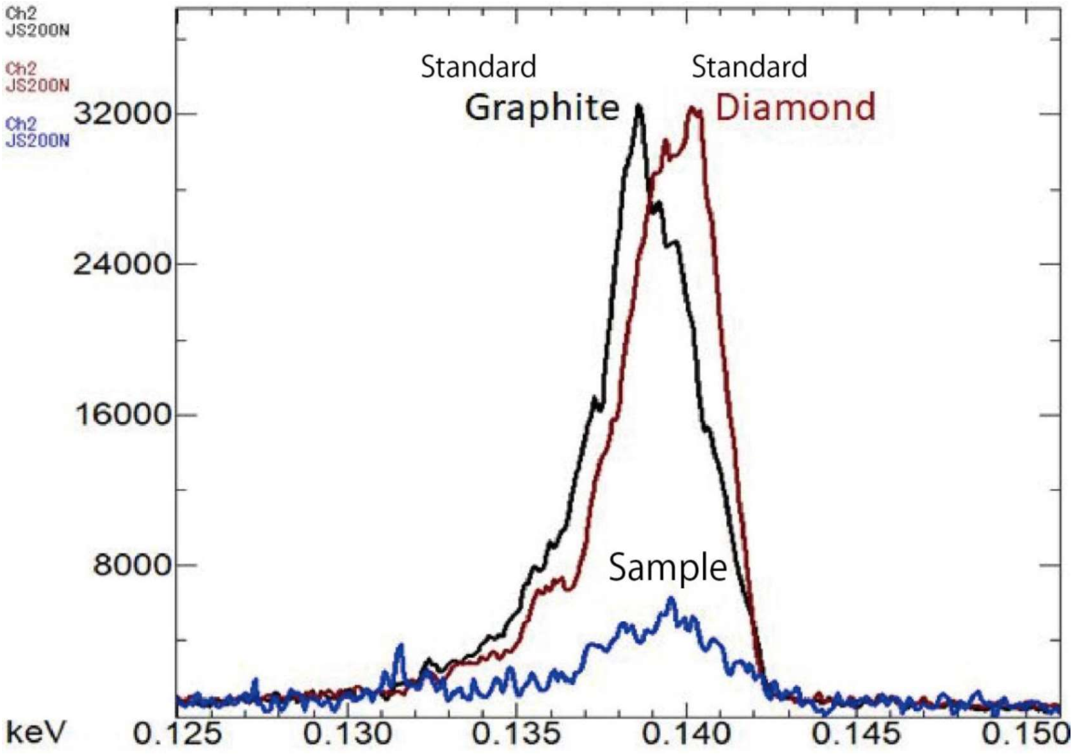

Supplementary Figure S3. EPMA-SXES spectrum of microdiamond (sample) compared with spectra of standard graphite and standard diamond. 2nd order C K-emission spectra of graphite (C-sp^2) and diamond (C-sp^3) show peaks at 138.5 eV and 140.0 eV, respectively. 2nd order C K-emission spectrum of the sample shows its peak close to C-sp^3.

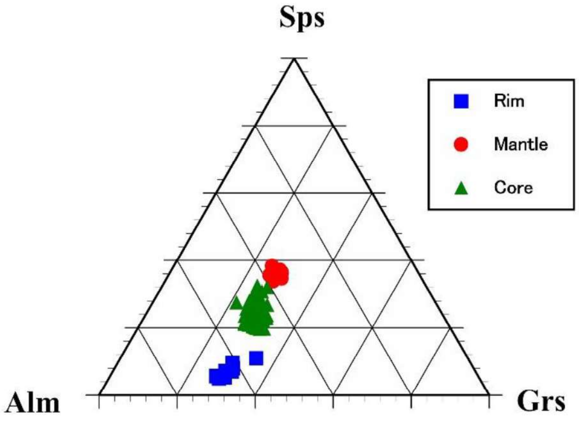

Supplementary Figure S4. Compositions of garnet from the garnetite in the spessartine (Sps) – almandine (Alm) – grossular (Grs) ternary diagram.

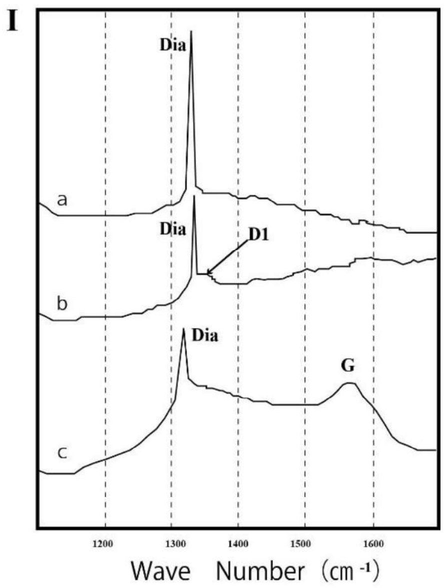

Supplementary Figure S5. Raman spectra of microdiamond with an arbitrary intensity scale. (a) Typical diamond (Dia) band at 1332 cm^{-1}. (b) Diamond band at 1332.5 cm^{-1} with a weak and broad D1 band of graphite. (c) Diamond band at 1316 cm^{-1} with a broad G band of graphite at around 1570 cm^{-1}.

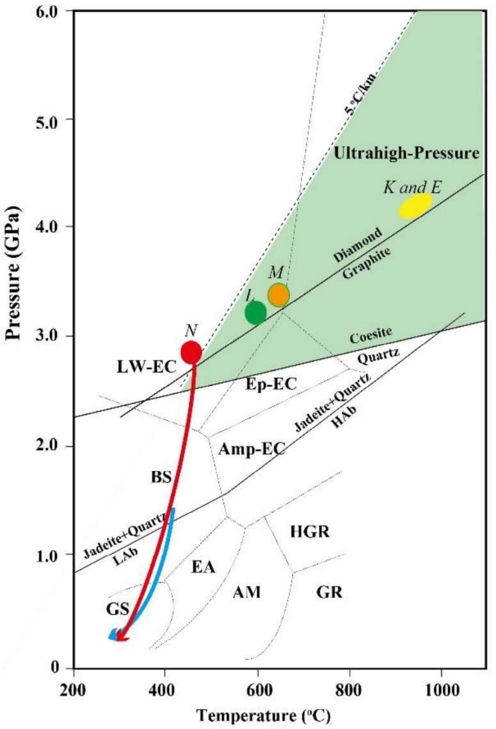

Supplementary Figure S6. Pressure-temperature (*P-T*) conditions for microdiamond formation in the Nishisonogi unit, which represents the lowest temperature among the worldwide UHP terranes. Colored circles and an ellipsoid indicate *P-T* conditions for some diamond-bearing UHP terranes. N: Nishisonogi unit (this study), L: Lago di Cignana[16], M: Maksyutov[42] (south Ural), K: Kokchetav[5], E: Erzgebirge[11]. Metamorphic facies boundaries are after Liou et al.[2,4]. Solid arrows indicate the possible *P-T* trajectory for the diamond-bearing metapelites in the Nishisonogi unit (red) and that for jadeitite[19] in the Nishisonogi unit (blue).
